# Deep Learning Based Computer-Aided Detection of Prostate Cancer Metastases in Bone Scintigraphy: An Experimental Analysis

**DOI:** 10.3390/jimaging12030121

**Published:** 2026-03-11

**Authors:** Eslam Jabali, Omar Almomani, Louai Qatawneh, Sinan Badwan, Yazan Almomani, Mohammad Al-soreeky, Alia Ibrahim, Natalie Khalil

**Affiliations:** 1 Department of Nuclear Medicine, King Hussein Medical Center, Royal Medical Services, Amman 11855, Jordan; 2 Department of Networks and Cybersecurity, Faculty of Information Technology, Al-Ahliyya Amman University, Amman 19111, Jordan; 3 Department of Clinical Oncology, Military Cancer Center, King Hussein Medical Center, Royal Medical Services, Amman 11855, Jordan; 4 Department of Internal Medicine, King Hussein Medical Center, Royal Medical Services, Amman 11855, Jordan

**Keywords:** cancer, bone scintigraphy, deep learning, convolutional neural networks (CNNs), computer-aided detection (CAD)

## Abstract

Bone scintigraphy is a widely available and cost-effective modality for detecting skeletal metastases in prostate cancer, yet visual interpretation can be challenging due to heterogeneous uptake patterns, benign mimickers, and a high reporting workload, motivating robust computer-aided decision support. In this study, we present an experimental evaluation of fourteen convolutional neural network (CNN) architectures for binary metastasis classification in planar bone scintigraphy using a unified protocol. Fourteen models, CNN (baseline), AlexNet, VGG16, VGG19, ResNet18, ResNet34, ResNet50, ResNet50-attention, DenseNet121, DenseNet169, DenseNet121-attention, WideResNet50_2, EfficientNet-B0, and ConvNeXt-Tiny, were trained and tested on 600 scan images (300 normal, 300 metastatic) from the Jordanian Royal Medical Services under identical preprocessing and augmentation with stratified five-fold cross-validation. We report mean ± SD for AUC-ROC, accuracy, precision, sensitivity (recall), F1-score, specificity, and Cohen’s κ, alongside calibration via the Brier score and deployment indicators (parameters, FLOPs, model size, and inference time). DenseNet121 achieved the best overall balance of diagnostic performance and reliability, reaching AUC-ROC 96.0 ± 1.2, accuracy 89.2 ± 2.2, sensitivity 83.7 ± 3.4, specificity 94.7 ± 2.2, F1-score 88.5 ± 2.5, κ = 0.783 ± 0.045, and the strongest calibration (Brier 0.080 ± 0.013), with stable fold-to-fold behaviour. DenseNet121-attention produced the highest AUC-ROC (96.3 ± 1.1) but exhibited greater variability in specificity, indicating less consistent false-alarm control. Complexity analysis supported DenseNet121 as deployable (~7.0 M parameters, ~26.9 MB, ~92 ms/image), whereas heavier models yielded only limited additional clinical value. These results support DenseNet121 as a reliable backbone for automated metastasis detection in planar scintigraphy, with future work focusing on external validation, threshold optimisation, interpretability, and model compression for clinical adoption.

## 1. Introduction

Prostate cancer is a very commonly diagnosed type of cancer in men, and it is one of the leading causes of death due to cancer in the world. As reported by Global Cancer Observatory (GLOBOCAN 2020), it causes over 1.4 million cases in a year and about 375,000 deaths in a year [1]. The skeletal system is the most frequent location of metastatic spread, and early detection of bone metastases is vital to proper staging, assessment of prognosis, and therapeutic planning [2,3,4]. The most popular imaging method of identifying skeletal metastases, due to its high sensitivity and the ability to cover the whole body, is bone scintigraphy [5], usually performed with technetium-99m-labelled phosphates. But manual interpretation of bone scans is time-consuming and subject to interobserver variation, leading to inconsistent clinical decisions. The mentioned issues have inspired the application of artificial intelligence (AI), machine learning, and deep learning algorithms in the workflow of oncologic imaging to provide automated, reproducible, and high-quality diagnostic assistance [6,7,8].

Bone scintigraphy provides an image of osteoblastic activity, and in metastatic lesions, this activity is high, and thus, similar patterns of uptake can also be caused by benign degenerative or inflammatory activity [9]. The malignant and non-malignant uptake regions are similar to each other; hence, their differentiation becomes challenging, especially for less experienced radiologists [10]. In addition, the increasing amount of imaging studies in the field of oncology has also placed more workload on radiologists, and it is necessary to have quality computer-aided diagnosis (CAD) systems that can help in the detection and classification of abnormal patterns [11]. One subgroup of deep learning algorithms, the convolutional neural networks (CNNs) [12,13], which are trained to extract hierarchies of spatial gradients of visual features directly from raw image data, has demonstrated outstanding ability to be successfully used in medical imaging, such as cancer detection, lesion localisation, and anomaly detection. CNNs, unlike the traditional machine learning methods [14], which rely on handcrafted feature extraction methods, automatically learn hierarchical features that capture both local and global context, enhancing generalisation and diagnostic reliability.

Over the last ten years, CNN architecture has advanced at an accelerated pace, and significant advancements have been made in image-based diagnosis [15]. The visual recognition deep learning architectures of AlexNet and VGG introduced the principles of deep learning that have been developed through stacked convolutional and pooling layers [16]. Later innovations, such as the addition of residual connections in ResNet [17], addressed these drawbacks and enabled significantly deeper networks to find good solutions. The architecture of WideResNet, which was built upon this concept, further expanded the network width to augment its representational power without incurring unreasonable depth, making it especially powerful with smaller or imbalanced medical datasets. The DenseNet architecture added dense connectivity [18], which encourages the sharing of features and efficient flow of gradients, and attention-enhanced variants like ResNet50Att and DenseNet121Att focus on clinically relevant regions to enhance localisation and interpretability. All these developments indicate that deep learning will keep transforming image-based diagnostics, at least in nuclear medicine.

Although there is considerable advancement in the automated process of identifying prostate cancer bone metastases through whole-body bone scintigraphy, there are still considerable gaps in the literature. Numerous studies consider single models or heterogeneous pipelines, which do not allow the architecture to be compared in identical conditions. Moreover, the use of one train-test split is still frequent, and this may cause sampling bias and lower generalizability [19]. Reporting performance is also frequently limited to accuracy or AUC, and even fewer studies use clinically and statistically informative metrics, like sensitivity/specificity and Cohen’s kappa (k), to measure agreement that is not by chance. All these restrictions indicate the necessity of a single, repeatable framework that subjects various architectures to a systematic benchmark under the same preprocessing, training, and validation conditions.

Although the current development of deep learning in prostate cancer imaging is very rapid, the evidence of computer-aided detection out of planar bone scintigraphy is still scattered and the reported performance results tend to be based on a difference in post-processing, post-processing augmentation, and optimisation parameters, and evaluation framework as opposed to actual benefits in architecture, which limits reproducibility and impedes clinically meaningful comparison. In addition, most studies emphasise peak discrimination measures, including the misrepresentation of reliability behavioural indicators such as sensitivity and specificity, agreement beyond chance (k-Cohen), and probability calibration (Brier score), which are needed to support safe decision -making in the normal operation of nuclear medicine processes. Correspondingly, this article does not introduce a new CNN architecture, but rather, a novel, clinically motivated and reproducible benchmark that quantitatively (systematically) measures trade-offs across representative CNN families under a single standardised protocol to offer practical guidance toward deployment oriented model selection, answering the research question of which CNN architecture provides the most desirable balance between AUC-ROC, clinical reliability (sensitivity/specificity and k) and calibration quality in prostate cancer bone metastasis classification of whole-body planar scintigraphy.

This proof-of-concept study provides an internally validated and reproducible benchmark of CNN architectures for prostate cancer metastasis detection on planar bone scintigraphy. Although the unified protocol establishes defensible baseline comparisons, multi-centre external validation across institutions, scanners, and acquisition protocols is required before any clinical deployment. In this work, clinical reliability refers to internally validated measures of discrimination and performance measures, including sensitivity/specificity, agreement (Cohen’s κ), and probability calibration (Brier score). Post hoc interpretability validation using explainable AI (XAI) was not performed in the present benchmark and remains a key direction for future work to confirm clinically plausible model attribution and strengthen clinical trust. The main contributions are as follows:

- Comprehensive architectural benchmark: A total of 14 CNN models are analysed and divided into diverse families: a baseline CNN (BaselineCNN); traditional architectures (AlexNet with batch normalisation, VGG16, and VGG19); residual networks (ResNet18, ResNet34, ResNet50, and ResNet50 with CBAM attention); dense networks (DenseNet121, DenseNet121 with CBAM attention, and DenseNet169); and modern architectures (WideResNet50_2 and EfficientNet-B0).
- Unified experimental guidelines: All the models are trained and tested within the same preprocessing, augmentation, and optimisation parameters to achieve equal comparison and reproducibility.
- Strong validation: The stratified five-fold cross-validation framework is used to reduce the sampling bias and give fold-convergent performance estimates.
- Clinically oriented, multi-metric evaluation: The metrics are AUC-ROC, accuracy, precision, recall/sensitivity, F1-score, specificity, and Cohen’s k, with probability-quality evaluation (Brier score) to put deployability into context.
- We provide a standardised set of validated baselines and an evidence-based discussion of architecture trade-offs, establishing a reference point for future extensions and supporting clinically feasible model selection for CAD deployment.

The rest of this paper will be structured as follows. Section 2 provides a literature review of related works in the field of prostate cancer imaging and deep learning-based analysis of bone scintigraphy. Section 3 discusses Materials and Methods. Section 4 provides a more general discussion of the results and clinical implications. Lastly, Section 5 provides a conclusion to the study and outlines possible directions for future research.

## 2. Related Work

Medical imaging use in the management of prostate cancer (PCa) has grown dramatically in the last decade due to the growth of imaging technologies and the growing accessibility of image analysis computational approaches [10,20]. Techniques like planar bone scintigraphy, SPECT/CT, MRI, and PET-based modalities have been at the centre stage in staging, treatment planning, and disease monitoring, especially in the detection of skeletal metastases, which is a significant cause of morbidity in advanced PCa [21]. With the increasing imaging volumes and the complexity of their interpretation, a significant amount of literature has developed, which has both synthesised the existing imaging knowledge and automated tools to facilitate clinical decision-making.

Across recent surveys [22,23,24,25,26,27,28,29,30], ML and DL are shown to support many PC imaging tasks, especially in mpMR and bpMRI, such as segmentation, lesion detection and characterisation, and PI-RADS assistance. Nuclear medicine evidence (PSMA PET) similarly indicates high accuracy for detecting metastatic disease and lymph node involvement, along with added capabilities such as tumour-burden quantification and workflow automation, although sensitivity can vary widely across studies. Despite these promising results, the literature repeatedly highlights major barriers to clinical translation: small, heterogeneous datasets; inconsistent reference standards; lack of standardised evaluation protocols; limited cross-validation; and scarce multicentre and external validation, together with the need for interpretability and clinically meaningful benchmarking. The aim of this section is to examine recent deep learning-based techniques that have been specifically designed to detect and classify prostate cancer, with particular interest in their model architectures, datasets, and reported performance.

Abbasi et al. [31] proposed a deep learning platform for prostate cancer detection using transfer learning, a pre-trained GoogleNet convolutional neural network. The research used prostate MRI images from a publicly available dataset at the Harvard Medical School, which consisted of prostate cancer and brachytherapy scans. The CNN model was trained to learn discriminative imaging features directly on the data and was evaluated compared to several conventional machine learning classifiers, like decision trees, Naive Bayes, and various variants of support vector machines, which used handcrafted features such as texture descriptors, morphological features, entropy measures, SIFT features, and elliptic fourier descriptors. The experimental outcomes, measured by sensitivity, specificity, accuracy, and ROC-AUC, revealed that the transfer learning-based CNN significantly outperformed the conventional machine learning methods, and that the deep feature representation of manual feature extraction is superior. Even though the performance was high as reported, there was a limitation of the study in that it only relied on a single architecture and dataset, and further comparative experiments on a range of deep learning models and standardised validation protocols should be considered.

In the meantime, Iqbal et al. [32] have made a thorough comparative analysis of deep and traditional machine learning methods of detecting prostate cancer based on the prostate MRI images. The authors examined handcrafted feature-based methods, such as texture, morphological, and GLCM features, used in combination with standard classifiers, such as SVM, KNN-Cosine, decision trees, RUSBoost, and kernel Naive Bayes, as well as deep learning models, based on LSTM networks and a transfer learning-based ResNet-101 architecture. Performance was evaluated using a publicly available MRI dataset and a strict 10-fold cross-validation protocol, and assessed using various measures, including accuracy, sensitivity, specificity, MCC, and ROC-AUC. The findings revealed that although the traditional classifiers yielded high performance when combined with optimised GLCM features, deep learning models, especially ResNet-101, were more consistent and consistently outperformed the conventional ones, achieving near-perfect classification accuracy and AUC values approaching 1.0. Regardless of these impressive performances, the research was based on a single deep residual architecture and a single MRI modality, which prevented generalisation and underscored the need for systematic benchmarking of various CNN architectures across distinct imaging tasks, which aligns with the comparative framework adopted by the current work.

Hosseinzadeh et al. [33] suggested the use of a deep learning-based computer-aided diagnosis (DL-CAD) system to detect and localise clinically significant prostate cancer (csPCa) by using bi-parametric MRI (bpMRI), specifically the effect of the size of the training data and the use of prior anatomical knowledge. The research involved a large multi-institutional cohort of 2734 biopsy-naive patients and developed a two-stage cascaded CNN system comprising a multi-planar anisotropic 3D U for prostate zonal segmentation and a U-Net-based lesion detection network. The authors have shown that both factors significantly affect diagnostic performance by systematically varying the size of the training dataset and incorporating zonal segmentation as prior knowledge. The proposed DL-CAD attained an AUC of 0.85 on an independent external test set used to detect histopathologically confirmed PCa, and lesion-level sensitivities were 87% when one false positive was allowed per patient. The comparative analysis demonstrated that the model was close to the expert radiologist, with moderate consistency between the DL-CAD and clinical results, as indicated by Cohen’s kappa. Notably, the paper emphasised that it takes significantly more than 2000 training cases, together with the introduction of domain-specific anatomical priors, to achieve near-expert-level performance, underscoring the need for large-scale data and structured knowledge integration to ensure reliable deep learning-based prostate cancer detection.

Han et al. [34] suggested and justified deep learning-related convolutional neural network (CNN) designs to identify bone metastases in prostate cancer patients using planar whole-body bone scintigraphy. The study sample comprised a large retrospective data set (9133 bone scans from 5342 patients with prostate cancer), categorised as metastatic or non-metastatic based on professional clinical reports and a review of the images. Two lightweight two-dimensional CNNs were constructed: a whole-body (WB) model that applies anterior–posterior scans and a global–local unified emphasis (GLUE) model that combines whole-body images with local patch-based information to identify both global skeletal patterns and focal lesions. Both abundant-data (72% training) and limited-data (10% training) models were tested on rigorous cross-validation. The findings showed great diagnostic accuracy in the WB model, with the AUC ranging from 0.933 to 0.957, and in the GLUE model, AUCs ranging from 0.936 to 0.955, depending on the amount of training data. The paper emphasised the benefits of lightweight CNNs and patch-based feature integration for planar nuclear medicine images, and that deep learning systems can be implemented within the commonly used clinical interpretation of bone scans. Nevertheless, the study was centred on the custom CNN architecture rather than on benchmarking of standard deep residual networks, so comparative research among popular CNN backbones, as in the current study, is necessary.

Mehralivand et al. [35] proposed and tested an entirely automated deep learning-based artificial intelligence model allowing biparametric MRI lesion detection of prostate cancer using large-scale and multi-institutional data and histopathological ground truth. The researchers used two pre-existing 3D convolutional architectures, U-Net and Anisotropic Hybrid Network (AH-Net), which were trained on MRI scans of 525 patients in two hospitals, and lesion-level predictions were based on MRI-TRUS-guided biopsies. It was trained on any ISUP grade of lesion, one of prostate cancer lesions, and tested on the lesion and patient level in terms of sensitivity, positive predictive value, Dice similarity coefficient, and false-positive rates. At the patient level, both architectures showed a patient-level detection sensitivity greater than 90. The sensitivities were better than AH-Net, which had better specificity and reduced false-positive rates than U-Net. At the lesion level, AH-Net again performed better compared to U-Net on the validation cohorts, with a sensitivity of 74.4% with fewer false positives per patient. Despite high performance, the authors have noted the existence of chronic problems of false-positive identification issues and highlighted that the system is more effective as an assistant tool than a fully autonomous diagnostic system. The article underscores the importance of deep learning in prostate MRI interpretation and the necessity of architecture-cognizant benchmarking and modality-definite evaluation, which directly inspires the comparative CNN analysis conducted in the current paper on planar bone scintigraphy.

Bosma et al. [36] proposed a new semi-supervised learning (SSL) framework of deep learning-based clinically significant prostate cancer detection with biparametric MRI, and tried to significantly limit the use of labour-intensive voxel-level annotations. The suggested approach is called report-guided SSL (RG-SSL), whereby routinely retrieved radiology reports are utilised and used to induce weak clinical supervision (the number of PI-RADS 4 lesions), which is then employed to create pseudo-labels for the unlabeled MRI tests. The experiment was conducted on a large multi-institutional cohort of 7756 MRI scans from 6380 patients, and external validation was done on a separate dataset of 300 patients with histopathological ground truth. RG-SSL always achieved better diagnostic performance than fully supervised learning and state-of-the-art SSL methods, even with limited annotation budgets, reaching an AUC of 0.86 ± 0.01 with only 100 manual labels, and at par with state-of-the-art supervised models trained on over 3000 labelled cases. Notably, the suggested methodology minimised annotation workload up to 14 times without affecting detection accuracy. Despite the limitations of the study on MRI-based lesion detection and the need to use structured clinical reports, it made clear that incorporating weak clinical knowledge into deep learning pipelines was effective, an important direction for scalable and annotation-efficient systems for detecting prostate cancer.

Talaat et al. [37] suggested an advanced deep learning system to detect prostate cancer by training a modified version of the ResNet50 architecture with Faster R-CNN and Mask R-CNN modules to improve the detection rate and location of the cancerous area in prostate MRI scans. The proposed Prostate Cancer Detection Model (PCDM) will use residual learning to achieve excellent feature extraction, candidate lesion localisation based on region proposal networks, and a dual-optimiser training regime to use Adam and stochastic gradient descent to balance feature generalisation and convergence speed. The model was trained and tested on a large dataset of approximately 11,000 MRI images with an 80/20 train-test split and evaluated against conventional diagnostic measures such as accuracy, sensitivity, specificity, precision, and F1-score. The results of the experiments showed that the PCDM based on ResNet50 performed better than the baseline ResNet50 and VGG architectures, with accuracies of 95.24, 97.40, 97.09, and 97.56, and sensitivities, specificities, and precisions of 97.56, 97.40, and 97.09, respectively. The research also incorporated ablation experiments to examine the effects of architectural depth, activation functionality, and optimiser settings, which revealed advantages of deeper residual models and two-optimiser training. Although reported results were strong, the study involved a single customised architecture and MRI modality, which further underscores the need for systematised benchmarking of various standard CNN and residual architectures and imaging modalities, which the current comparative study aims to achieve directly.

Korb et al. [38] examined the possibility of local prostate cancer recurrence detection with the help of deep learning based on [18F]-PSMA-1007 PET/CT imaging in a difficult clinical post-treatment setting. The researchers used 1404 PET/CT scans of 1145 patients with histologically proven prostate cancer to study the relationships between these variables retrospectively and created numerous CNN models based on DenseNet121 architecture deployed in the MONAI framework. A variety of model variants were systematically tested, including whole-body analysis, prostate-focused spatial cropping with automated segmentation of organs, the use of clinical metadata (prostatectomy status and PSA levels), and the use of extensive data augmentation with hyperparameter optimisation. Although the performance gradually increased through progressive refinement, the highest -performing model had a validation accuracy of 77.1, balanced accuracy of 73.9, and AUC of 0.75, with lower sensitivity was found on an independent test set. Notably, the authors noted a significant overfitting and determined that an appropriate dataset size was insufficient, despite the advanced modelling techniques used to obtain a clinically acceptable level of accuracy. The study presents useful negative and repeatable evidence that it is intrinsically challenging to detect local recurrence by PET/CT, and that larger datasets, specific modality optimisation, and other architectural designs are required. These results also support the significance of systematic inter-model and inter-imaging benchmarking, which is the direction the current research takes.

In summary, the deep learning research highlighted in Table 1 shows that CNN-based structures can achieve great success in detecting prostate cancer in MRI and PET/CT, but the research on planar bone scintigraphy is relatively scarce-bones scans are actively employed in the metastatic staging. Nevertheless, the current literature continues to highlight several recurring gaps that drive this work. To begin with, most of the published work is modality-based, with most focusing on MRI; there are few studies that give dedicated and architecture-level analysis on whole-body planar scintigraphy. Second, a lot of papers only consider a single (and sometimes two) DL architecture, which can be custom-designed or massively modified backbones, which complicates the separation of the architecture choice effect and dataset-specific and implementation-specific factors. Third, reporting can often be limited to headline metrics (e.g., accuracy and AUC), whereas clinically meaningful and operational measures (e.g., Cohen’s k (agreement beyond chance) vs. specificity trade-offs and deployment-oriented complexity measures, e.g., model size and inference time) are often not reported. Lastly, strict and reproducible validation is not always performed; many studies have used single splits, which limit comparability and generalizability. To overcome these shortcomings, we introduce a set of 14 standardised, reproducible baseline CNN architectures (classic, residual, dense, attention-augmented, and modern backbones), trained under the same preprocessing/augmentation and optimisation conditions and evaluated by a stratified five-fold cross-validation protocol with an internal validation split at each of the five folds. In addition to traditional classification metrics, we report AUC-ROC, sensitivity, specificity, precision, F1-score, Cohen’s kappa, and Brier score, as well as deployment-related complexity (parameters, FLOPs, model size, and latency), along with 95% confidence intervals. Generally, this framework provides clinically congruent evidence that can be applied to accuracy, reliability, and efficiency trade-offs, to inform and guide the choice of models for high-throughput nuclear medicine workflows.

Although all previous studies demonstrate the promise of DL for PC imaging, evidence in planar bone scintigraphy is still fragmented due to variations in preprocessing, training of the model, and design of evaluation. This makes it challenging to specify whether performance differences reflect true architectural or protocol-dependent effects. In addition, clinically meaningful aspects such as reliability and calibration are not always reported, despite being critical for decision support. These gaps motivate a controlled benchmark under a unified protocol that evaluates architecture using discrimination, reliability, and calibration criteria.

## 3. Materials and Methods

**Figure 1:**
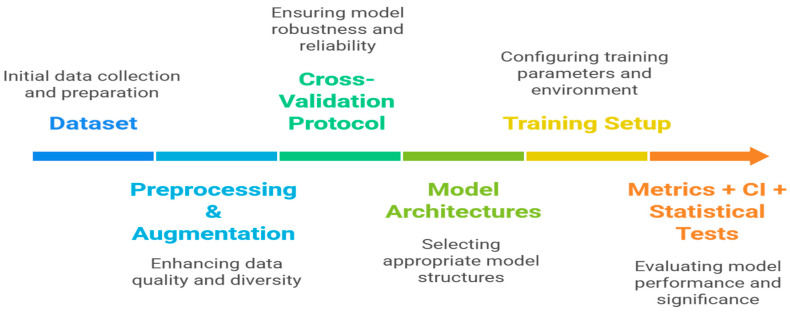
Methodological framework flowchart.

This section explains the methodological paradigm that was used to compare the evaluation of deep residual and convolutional architectures for detecting prostate cancer using bone scintigraphy. To make a fair and reproducible cross-architectural comparison, all experiments were run with standardised preprocessing, data splitting, and training settings. A 5-fold cross-validation approach was used to evaluate the performance of generalisation and reduce the impact of sampling bias. The methodological framework flowchart is presented in Figure 1.

### 3.1. Data Source, Ethics Approval, and Dataset Description

In this study, a personal collection of clinical bone scintigraphy data of the Royal Medical Services of Jordan (JRMS) was utilised. The IRB, the JRMS Institutional Review Board, granted ethical approval for the study protocol. All images were processed in accordance with the laws of the JRMS and ethical norms, and the patient’s identification was removed before processing. Since the dataset is confidential and includes clinical information, it is not publicly accessible; access can be considered with JRMS and proper approvals.

This study included prostate cancer (PC) patients who were referred to the Nuclear Medicine Department at JRMS to carry out bone scintigraphy in the period between 2018 and 2022. The sample consisted of 600 whole-body planar bone scintigraphy images, which were obtained by anterior and posterior orientation following administration of Technetium-99m methylene diphosphonate (Tc-99m MDP). Each patient provided one image (one scan record). The dataset was balanced with 300 normal and 300 abnormal scans. The metastatic-positive cohort of 300 cases was distributed as follows: 75 cases (25.0%) had solitary metastatic involvement, whereas 225 cases (75.0%) presented with multiple metastatic lesions. In terms of anatomical distribution, 103 cases (34.3%) involved the axial skeleton only, 23 cases (7.7%) involved the appendicular skeleton only, and 174 cases (58.0%) involved both axial and appendicular regions. Ground-truth labelling was established based on clinical follow-up in 60% of metastatic-positive cases and confirmatory MRI/CT in 40% of cases.

**Figure 2:**
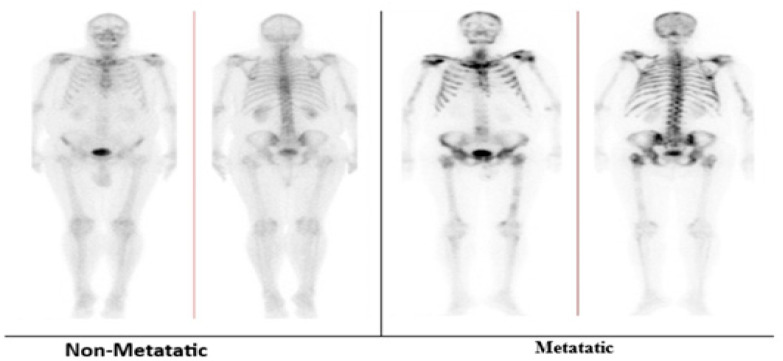
Dataset sample of normal healthy bone and abnormal metastatic cases.

A nuclear medicine physician consultant assigned ground-truth labels biassed on clinical interpretation. The deviant class was associated with the established bone metastasis, and the normal class was associated with the scans that did not show any indication of bone metastasis in the bone scintigraphy. These labels served as a training and evaluation reference point for the deep learning classifiers. The metastatic class was defined as any bone metastasis attributable to PC, irrespective of metastatic burden; therefore, scans with solitary or multiple metastatic lesions were labelled as metastatic-positive. Ground truth labels were confirmed using follow-up imaging and oncology records as the clinical reference standard. An example of normal bones and metastatic bones is presented in Figure 2.

### 3.2. Image Preprocessing, Augmentation, and Cross-Validation Protocol

All the bone scintigraphy images were loaded in their original formats and converted to RGB to be compatible with the standard CNN architectures [39]. All the images were rescaled to a constant 224 × 224 pixel spatial resolution using bilinear interpolation [40], which corresponds to the input dimensions of ImageNet-pretrained backbones and provides uniform input across all architectures being tested. Normalisation of pixel intensities was done based on ImageNet statistics (mean = [0.485, 0.456, and 0.406], standard deviation = [0.229, 0.224, and 0.225]) that puts the input distribution within the range of the distribution over which the pretrained weights are optimised (when enabled) and enables optimisation to be stable [41].

**Figure 3:**
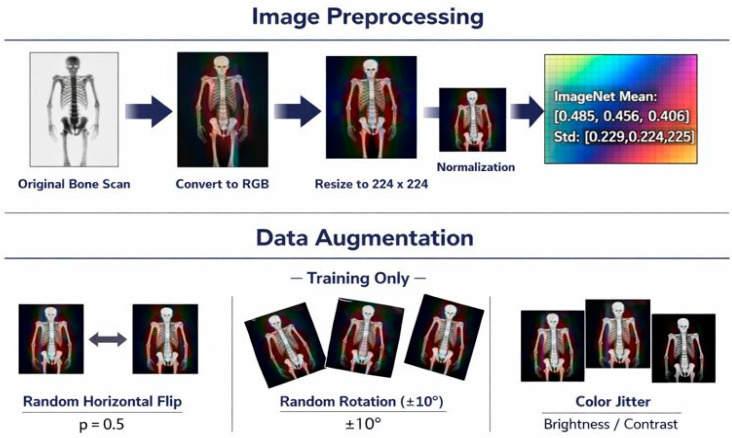
Image preprocessing and augmentation operations.

To improve model generalisation and reduce overfitting, data augmentation was only done to the training subset in cases of every cross-validation fold. The augmentation pipeline comprised the following: first, a random horizontal flipping (probability = 0.5), second, random rotation in ±10 deg, and lastly, colour jittering (brightness = 0.2, contrast = 0.2) [42]. Validation and test images were run without augmentation, with only resizing and normalisation, which ensures that the performance on untouched clinical images was evaluated. Figure 3 shows exemplary images from the found image preprocessing and training-time augmentation processes.

**Figure 4:**
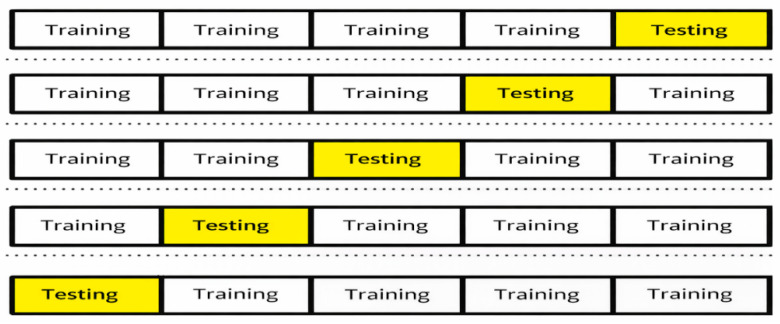
Five-fold cross-validation.

To give a good estimate of the performance, a stratified 5-fold cross-validation protocol was used, such that the original class distribution (normal vs. abnormal) is maintained in all the splits [43]. Samples were divided into a training set (approximately 80 percent) and a held-out test set (approximately 20%) in every fold, as in Figure 4. A stratified split was then used to form the training set and validation sets, with 20% of the set going to validation, resulting in a rough split of 64% training, 16% validation, and 20% test per fold. The design ensures disjoint train/validation/test sets within each fold and is compatible with early stopping and best-checkpoint selection based on validation performance.

### 3.3. Deep Learning Model Architectures

We have comprehensively benchmarked DL performance for detecting PCa bone metastases in planar scintigraphy using 14 CNN models based on classic, residual, densely connected, and modern efficient backbones. All the models were trained in PyTorch, and where possible, the network was trained on torchvision backbones by replacing the original classification head with a fully connected layer that produced two logits (normal vs. abnormal).

#### 3.3.1. CNN

CNN was developed as a lightweight to give a clear reference architecture. It is composed of four convolutional blocks with the format of (Conv2D-Batch Normalisation-ReLU-MaxPooling) and number of channels that are used in gradually increasing (32-64-128-256) [44]. Spatial features are then represented in a 256-dimensional representation using a global adaptive average pooling layer, then a dropout-regularised dropout-to-256-to-128-to-ReLU-to-128-to-2 classifier (Dropout 0.5-FC 256-128-ReLU-Dropout 0.3-FC 128-2). The focus of this structure is on simplicity, a few parameters, and stable optimisation, with sufficient capacity to achieve binary discrimination.

#### 3.3.2. AlexNet

AlexNet was also added as a classical deep CNN baseline and improved by adding batch normalisation to the end of each convolutional layer to increase the stability and convergence of training [45]. It is a network based on a canonical multi-stage convolutional architecture with large receptive fields in the early stages, using convolution blocks that are repeated, followed by an adaptive average pooling layer (6 × 6), which is used before the fully connected stack. The classifier still applies to the high-capacity standard design (two 4096-unit FC layers), but then it is ultimately changed to binary classification with a 2-unit output layer.

#### 3.3.3. VGG16

The VGG16 is a plain CNN architecture [46,47], using 3 × 3 convolutions and periodic max pooling. It has the strength of utilising small, homogeneous kernels to construct depth-based features. In this case, the last classification layer of the VGG classifier stack was replaced with a 2-neuron layer that would allow direct classification of normal and metastatic scans and would maintain the feature extraction pipeline of VGG.

#### 3.3.4. VGG19

VGG19 is a version of VGG16 with extra convolutional layers [46], which adds depth and representational capacity without modifying the principles of its design (repeated 3 × 3 convolution blocks + max pooling). As with VGG16, the final classifier layer was substituted with a 2-unit output layer for binary classification, enabling evaluation of whether additional depth benefits scintigraphy metastasis recognition in planar scintigraphy.

#### 3.3.5. ResNet18

ResNet18 is a residual network architecture [48]. It has skip connections to allow stable gradient flow and overcome vanishing gradients. It consists of overlapping residual blocks of successively increasing feature channels at successive stages. The last FC layer was replaced with a 2-output FC head in case of binary classification, keeping the original residual feature extractor.

#### 3.3.6. ResNet34

ResNet34 enhances the residual design on top of ResNet18 with more residual blocks per stage, but uses the same skip-connection mechanism [48]. The effect of increased residual depth on the classification performance was evaluated using this model. The last FC layer was also replaced with a 2-output classifier.

#### 3.3.7. ResNet50

ResNet50 consists of bottleneck residual blocks (1 × 1-3 × 3-1 × 1), which add representational power but with a fixed computational cost [48,49]. The network generates a high-level feature tensor, which is pooled and fed through a final FC layer. In this experiment, the 2-class output head was used in place of the FC layer in the study to detect metastasis.

#### 3.3.8. ResNet50 (Attention Version) with CBAM Attention

To determine the advantage of attention-enhanced residual learning, a Convolutional Block Attention Module (CBAM) was added to ResNet50 [50]. CBAM was applied before global average pooling and was placed at the end of the final residual stage (high-level feature tensor with 2048 channels). The module uses channel attention (global average/max pooling + MLP gating) with spatial attention (7 × 7 convolution over aggregated channel statistics) to recalibrate the feature responses, thereby focusing on those that are diagnostically important. The classifier’s output was a 2-logit FC layer.

#### 3.3.9. DenseNet121

DenseNet121 [51] uses dense connectivity with every layer, taking the concatenated results of all the previous layers as part of a dense block. This encourages sharing of features and efficient gradient flow and can tend to learn with fewer parameters than effectively deep plain networks. A 2-unit linear head was used to replace the original classifier, keeping the DenseNet feature extractor.

#### 3.3.10. DenseNet121_Attention (CBAM Attention)

In the case of the attention-augmented DenseNet, a CBAM block was added to the last DenseNet feature (1024 channels) right before pooling and classification [52]. This final stage of attention will isolate the most abstract features with the amplification of informative channels and spatial areas. The last classifier was a 2-unit linear layer, which allowed making a direct comparison between attention vs. non-attention DenseNet121 in the same training conditions.

#### 3.3.11. DenseNet169

DenseNet169 [53] is a more profound DenseNet design that has additional layers and capacity that retain dense connectivity and feature reuse. It was added to test the effect of the deeper dense representations on the classification of metastasis. The last classifier was substituted with a 2-output linear layer.

#### 3.3.12. WideResNet50_2

WideResNet50-2 adds channels (width of blocks) to the residual blocks, compared with the complementary ResNet50, enhancing representational power without adding significant depth. Wider residual features were investigated using this model to determine whether they, in scintigraphy, can better discriminate against metastatic patterns. The last FC layer was replaced with a 2-class output head.

#### 3.3.13. EfficientNet-B0

EfficientNet-B0 is an efficient driven architecture [54,55], which balances network depth, width, and resolution. It internally has MBConv blocks, squeeze-and-excitation style channel recalibration, and can trade-off accuracy-efficiency well. To conduct this research, the last classification layer in the EfficientNet classifier module was substituted by a 2-class linear layer, which allows comparing the results on a similar preprocessing and CV protocol.

#### 3.3.14. ConvNeXt-Tiny

ConvNeXt-Tiny is a recent CNN architecture that uses design decisions inspired by transformers but uses convolutional operations. It employs redesigned convolutional blocks (e.g., larger kernel depthwise convolutions and better normalisation/activation positioning) to improve feature learning. The last classification head was substituted by a 2-class linear layer, making it possible to evaluate the modern CNN design effectiveness in planar bone scintigraphy classification.

### 3.4. Training Setup

Training was done for all the models with a common optimisation strategy to achieve equal comparisons across architectures. The objective of classification was a binary two-logit problem (normal vs. abnormal) and was reduced to using the cross-entropy loss. It was trained in PyTorch 2.9.1 and run on CPU or supported by GPU (CUDA). The AdamW Optimizer was used, which does not tie weight decay with the gradient-based changes on the parameters, and this enhances deep network generalisation. The learning rate was kept constant at 1 × 10^−4^, and weight decay was also kept at 1 × 10^−4^; the models were trained with a batch of 16 for 50 epochs per fold. Randomness was managed by seeding Python 3.13.5, NumPy 2.1.3, and PyTorch to control randomness to improve the reproducibility; with the presence of CUDA, the CUDA random seed was also seeded.

The training on the validation subset was observed after each epoch in each cross-validation fold. The choice of the model was based on the validation ROC-AUC, and the best model in terms of validation AUC in the fold was stored as the best-performing model in a fold. An early stopping mechanism was implemented with a patience of 10 epochs to reduce overfitting and unnecessary computation: the training was stopped in case of no improvement of the validation AUC in 10 consecutive epochs. The optimal checkpoint of each fold was then reloaded and tested on the appropriate held-out test subset to generate final fold-level predictions and probabilities.

### 3.5. Evaluation Metrics

**Figure 5:**
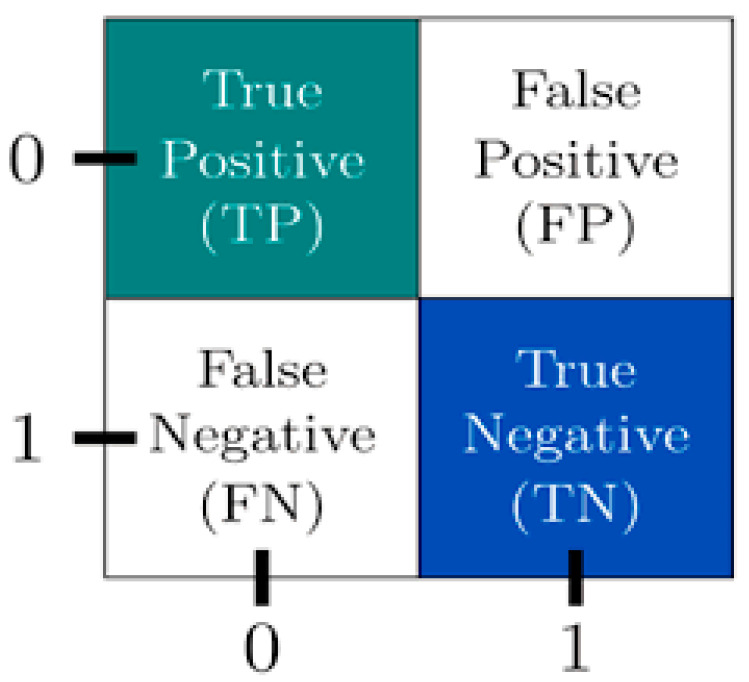
Confusion matrix.

Evaluating the outer test split of both hard class prediction and probabilistic output under each stratified cross-validation fold, Model Performance would be evaluated. Each test image in all the models generated two logits (normal vs. abnormal), which were converted to class probability through the softmax function. Argmax gave the predicted label, and probability-dependent evaluation was performed on the positive-class probability p(abnormal), e.g., ROC-AUC and Brier score. Confusion matrices (TP, FP, FN, and TN) were directly noted down to allow straightforward accuracy, precision, recall, F1, sensitivity, and specificity. The confusion matrix is indicated in Figure 5.

Each matrix is calculated as follows:

(1) $$\mathrm{Accuracy}=\frac{TP+TN}{TP+TN+FP+FN}$$

(2) $$\mathrm{Precision}=\frac{TP}{TP+FP}$$

(3) $$\mathrm{Recall}=\frac{TP}{TP+FN}$$

(4) $$F1=\frac{2\cdot \mathrm{Precision}\cdot \mathrm{Recall}}{\mathrm{Precision}+\mathrm{Recall}}$$

(5) $$\mathrm{Sensitivity}=\frac{TP}{TP+FN}$$

(6) $$\mathrm{Specificity}=\frac{TN}{TN+FP}$$

(7) $$\mathrm{ROC}–\mathrm{AUC}=\int_{0}^{1}\mathrm{TPR}(\mathrm{FPR})\;d(\mathrm{FPR})$$

where

(8) $$\mathrm{TPR}=\frac{TP}{TP+FN},\mathrm{FPR}=\frac{FP}{FP+TN}$$

(9) $$\mathrm{Brier}\;\mathrm{score}=\frac{1}{N}\sum_{i=1}^{N}(p_{i}-y_{i}{)}^{2}$$

where $p_{i}\;$is the predicted probability of the abnormal class and $y_{i}\in \left(0,1\right)\;$is the reference label.

(10) $$Cohen’s\;Kappa\;\left(\kappa \right)=\frac{p_{o}-p_{e}}{1-p_{e}}$$

where $p_{o}\;$ is the observed agreement and $p_{e}\;$is the agreement expected by chance.

All data were calculated on a per-fold basis with respect to the held-out test subset and were subsequently summarised on a fold-by-fold basis (descriptive statistics) (mean, standard deviation, minimum, and maximum), which gives strong estimates in repeated stratified resampling. To quantify the uncertainty along with point estimates, we have computed all the reported metrics of performance on 95% confidence intervals (CIs). Because the intended clinical use (screening/triage vs. staging/confirmation) determines acceptable false-positive and false-negative rates, decision thresholds should be selected accordingly. In this study, we report threshold-dependent reporting by presenting sensitivity and specificity alongside discrimination and calibration (including Brier score) under the unified protocol.

## 4. Experiments and Results

To answer the research question posed in the introduction section, a comparison of 14 CNN architectures under identical experimental settings and interpret performance using three complementary perspectives: discrimination (AUC-ROC), clinical reliability (sensitivity/specificity and Cohen’s κ), and probability calibration (Brier score). This allows identification of models that provide a favourable balance between detection performance and reliability, rather than relying on a single headline metric.

### 4.1. Setting of Experiments

The experiments were carried out on the workstation with Windows 11 (64-bit) and Intel Core i7 CPU (3.2 GHz), and 16 GB RAM. In Table 2, the general training configuration hyperparameter settings are indicated. The Python language was used to run all experiments.

Table 3 lists each architectural setting of CNN under study in this paper. The general structure of all models was different in their backbone connectivity patterns, along with selective inclusion of attention mechanisms, even though uniform training conditions (preprocessing, cross-validation, and hyperparameters) were upheld. These important architectural variables, together with the respective adjustments that have been carried out to the name of the classification head of each of the networks in the binary task, are tabulated.

### 4.2. Results and Discussion

This section provides a comprehensive analysis of the experimental results obtained by evaluating a range of convolutional, residual, dense, and attention-augmented deep learning architectures for detecting prostate cancer bone metastases from whole-body planar scintigraphy. All experiments were conducted under a unified stratified five-fold cross-validation protocol, ensuring that the reported outcomes capture both mean performance and fold-to-fold variability. For each model, we summarise discrimination and classification performance using AUC-ROC, accuracy, precision, recall (sensitivity), F1-score, specificity, and Cohen’s κ, and we further assess calibration quality via the Brier score. To contextualise predictive performance in terms of real-world feasibility, we additionally report computational indicators including parameter count, FLOPs, memory footprint (model size), and inference latency. Beyond descriptive comparisons, we incorporate statistical hypothesis testing to determine whether observed performance differences are significant rather than attributable to sampling variability, using fold-wise results to perform model ranking and pairwise comparisons where appropriate. The results are organised into five complementary perspectives: (i) Model Performance: Quantitative, presenting the primary predictive metrics; (ii) Clinical Reliability Analysis: Missed Metastasis vs. False Alarms, contrasting false-negative and false-positive behaviour to highlight clinically relevant safety trade-offs; (iii) Model Performance: Discrimination versus Calibration, evaluating both separability and probability reliability; (iv) Model Performance: Stability and Variance, examining robustness and consistency across folds; and (v) Model Performance: Complexity (Efficiency and Deployability), comparing computational costs and latency to inform practical deployment.

#### 4.2.1. Model Performance: Quantitative

**Figure 6:**
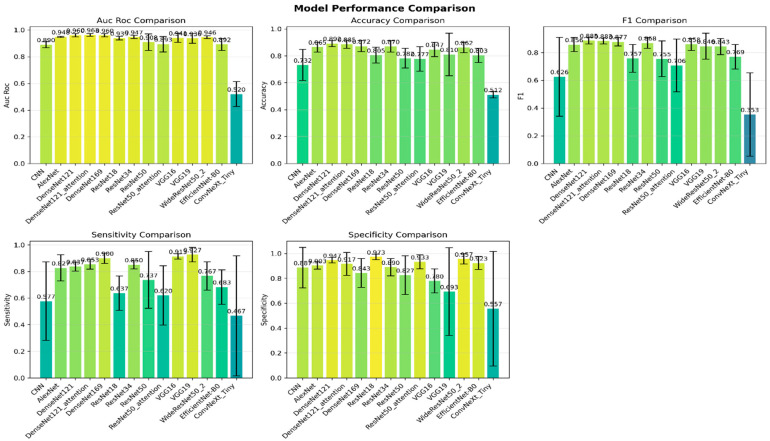
Model performance.

Across the stratified five-fold cross-validation, the tested architecture exhibited clear separation in overall discrimination and classification quality. Table 4 and Figure 6 summarise the obtained quantitative performance results (mean ± SD) for all 14 models, reporting AUC-ROC, accuracy, precision, recall (sensitivity), F1-score, specificity, and Cohen’s κ to enable a consistent side-by-side comparison.

The performance ranking in Table 4 can be interpreted in relation to planar bone scintigraphy, where discriminative cues are often weak, spatially diffuse, and confounded by benign uptake (e.g., degenerative changes). DenseNet121 provides the most consistent overall trade-off (AUC-ROC: 96.0 ± 1.2; Accuracy: 89.2 ± 2.2; κ: 0.783 ± 0.045) and the best calibration (lowest Brier score: 0.080 ± 0.013). This behaviour is consistent with DenseNet’s dense connectivity, which encourages feature reuse and stable gradient flow, allowing multiscale uptake patterns to be integrated across layers without relying on strong local texture cues. DenseNet169 increases sensitivity (90.0 ± 3.8) but shows reduced specificity (84.3 ± 11.7), suggesting a higher tendency toward false positives in the presence of benign mimickers. Attention augmentation (CBAM) yields non-uniform effects: DenseNet121 + CBAM slightly improves AUC and sensitivity (96.3 ± 1.1; 85.3 ± 3.6) but does not improve calibration (Brier: 0.085 ± 0.017), whereas ResNet50 + CBAM decreases performance compared with ResNet50 (AUC: 89.3 ± 5.7 vs. 90.8 ± 6.3; F1: 70.6 ± 18.9 vs. 75.5 ± 12.9), indicating that attention reweighting may amplify non-specific hotspots under image-level supervision and limited data. Among residual models, ResNet34 achieves a strong balance (AUC: 94.7 ± 1.3; F1: 86.8 ± 3.9), while ResNet18 shows very high specificity (97.3 ± 2.3) but lower sensitivity (63.7 ± 12.9), reflecting a more conservative decision boundary. Notably, ConvNeXt-Tiny performs near chance level (AUC: 52.0 ± 9.3; Accuracy: 51.2 ± 2.3; κ: 0.023 ± 0.047; Brier: 0.250 ± 0.002), suggesting poor alignment with scintigraphy’s low-texture intensity statistics and/or higher sensitivity to data scale and optimisation in this nuclear-medicine setting. Overall, architectures supporting robust global aggregation and stable feature propagation (DenseNet121, ResNet34) appear better matched to planar bone scintigraphy than attention variants or modern CNN designs without domain-specific adaptation. Across models, DenseNet121 provides the strongest overall balance (high AUC-ROC and κ with the lowest Brier score), whereas ConvNeXt-Tiny performs near chance level, indicating poor suitability under the current scintigraphy setting and protocol. Attention augmentation (CBAM) shows non-uniform effects across backbones, underscoring the importance of reliability and calibration reporting when selecting clinically deployable architectures.

These quantitative differences were also supported by statistical analysis. The global comparison across the 14 models showed a significant overall effect (Friedman χ^2^ = 39.67, *p* = 0.000156), confirming that the observed performance gaps are unlikely to be explained by fold-level sampling variability alone. The corresponding average rank ordering further aligned with the metric-wise findings, placing DenseNet121 (rank 3.0), DenseNet121-attention (3.6), and DenseNet169 (3.8) as the top-performing group, while the baseline CNN (11.2) and ConvNeXt-Tiny (14.0) ranked lowest.

#### 4.2.2. Clinical Reliability Analysis: Missed Metastasis vs. False Alarms

While aggregate metrics summarise overall performance, clinical usefulness is better reflected by how each model trades off missed metastasis (false negatives) against false-alarms (false positives). Based on the fold-wise sensitivity and specificity reported in Table 4, we quantify these clinically relevant risks as FNR = 100 − sensitivity (missed metastasis rate) and FPR = 100 specificity (false alarm rate). The resulting error profiles are summarised in Table 5, enabling a direct comparison of safety-critical behaviour across models.

Models prioritising the minimisation of missed metastasis achieved the lowest FNR values. The VGG variants were the most sensitivity-oriented, with VGG19 yielding the lowest missed metastasis rate (FNR 7.3% ± 5.4) and VGG16 similarly low (FNR 8.7% ± 1.9). However, this benefit was accompanied by substantially increased false alarms, particularly for VGG19 (low and highly variable specificity), translating into the highest FPR (30.7% ± 35.3). This pattern indicates an aggressive “rule-out” tendency—valuable when the clinical priority is not to miss metastatic disease, but potentially costly due to elevated downstream workload and unnecessary follow-up investigations.

Conversely, models optimised for minimising false alarms behaved more conservatively. ResNet18 achieved the lowest false alarm burden (FPR 2.7% ± 2.3) but at the expense of a much higher missed metastasis risk (FNR 36.3% ± 12.9), reflecting a decision strategy that favours high certainty for positive predictions while failing to capture a substantial fraction of true metastatic cases. A more clinically practical operating point was observed for the top DenseNet models, particularly DenseNet121, which offered a strong compromise between safety and workload by combining low FPR (5.3% ± 2.2) with robust detection (FNR 16.3% ± 3.4). The attention-augmented DenseNet121-attention slightly reduced missed metastasis (FNR 14.7% ± 3.6) but increased false alarms and exhibited greater variability in specificity, suggesting that attention shifts the decision boundary toward sensitivity without consistently preserving reliability across folds.

Overall, these findings emphasise that the best model depends on the intended clinical use-case: screening-oriented workflows may favour high-sensitivity models (e.g., VGG16/VGG19) to reduce missed metastasis, whereas workload-constrained settings may prefer models with lower false alarms (e.g., ResNet18/WideResNet50_2). Among all candidates, DenseNet121 provides the most balanced clinical profile in Table 5, achieving a favourable FNR–FPR trade-off that is well-suited for practical deployment where both safety and resource burden matter.

#### 4.2.3. Model Performance: Discrimination Versus Calibration

**Figure 7:**
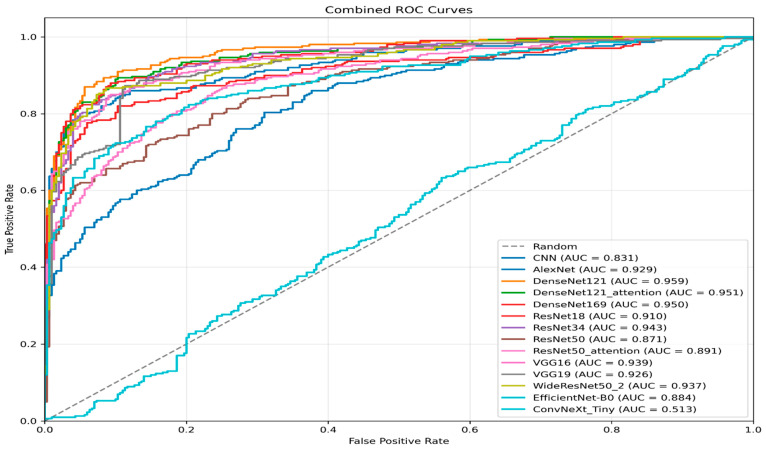
ROC curves.

Beyond conventional accuracy type metrics, a clinically deployable model should demonstrate both strong discrimination and the ability to separate metastatic from non-metastatic scans and reliable calibration, meaning that predicted probabilities correspond to true outcome likelihood. Table 6 summarises these complementary properties using AUC-ROC (discrimination) and the Brier score (calibration; lower is better), while the combined ROC curves in Figure 7 visually confirm the relative separability of the evaluated architectures.

In terms of discrimination, performance was led by the DenseNet family. DenseNet121-attention achieved the highest AUC-ROC (0.963 ± 0.011), closely followed by DenseNet121 (0.960 ± 0.012) and DenseNet169 (0.960 ± 0.011), a level commonly interpreted as excellent discrimination. A second tier of strong separability was observed for AlexNet (0.948 ± 0.004) and ResNet34 (0.947 ± 0.013), indicating that both classical and modern backbones can produce competitive ROC behaviour on this task. By comparison, the ResNet50 family showed weaker discrimination (ResNet50: 0.908 ± 0.063; ResNet50-attention: 0.893 ± 0.057), while the baseline CNN reached only moderate separation (0.890 ± 0.023). ConvNeXt-Tiny performed near chance level (0.520 ± 0.093), consistent with its ROC curve tracking the diagonal in Figure 7. Importantly, a global Friedman test across fold-wise AUC-ROC values confirmed statistically significant differences among the 14 models (χ^2^(13) = 39.67, *p* = 0.000156), supporting that the observed AUC gaps reflect systematic architectural effects rather than random fold variability.

Calibration analysis further showed that strong discrimination does not necessarily guarantee reliable probability estimates. The most favourable calibration was obtained by DenseNet121 (Brier 0.080 ± 0.013), followed by DenseNet121-attention (0.085 ± 0.017) and DenseNet169 (0.093 ± 0.033), suggesting that the top DenseNet models not only separate classes effectively but also output comparatively trustworthy probabilities. In contrast, several architectures with reasonable AUC exhibited weaker calibration, e.g., ResNet18 (0.144 ± 0.035) and the baseline CNN (0.172 ± 0.048), while ConvNeXt-Tiny showed the poorest calibration overall (0.250 ± 0.002). From a clinical decision-support perspective, where probability thresholds may guide follow-up recommendations, these results favour DenseNet121 as the most balanced choice, offering near-top discrimination together with the most reliable calibration among all evaluated models.

#### 4.2.4. Model Performance: Stability and Variance

Beyond mean performance, a clinically reliable system must exhibit stable behaviour across folds, because large fold-to-fold fluctuations can translate into unpredictable outcomes when deployed on new patient cohorts. Accordingly, Table 4 reports all metrics as mean ± SD under stratified five-fold cross-validation, enabling direct assessment of robustness in addition to peak accuracy. To make stability trends easier to interpret, Table 7 (Performance Stability Analysis) summarises each architecture’s Friedman average rank (derived from fold-wise results; global test significant with *p* = 0.000156) together with the most clinically relevant variance patterns, highlighting where instability concentrates (e.g., sensitivity or specificity) and the resulting clinical risk implications.

Overall, DenseNet121 demonstrated the most reliable high-performing behaviour, achieving strong averages with consistently low dispersion across key metrics (AUC-ROC 96.0 ± 1.2, accuracy 89.2 ± 2.2, sensitivity 83.7 ± 3.4, specificity 94.7 ± 2.2, Brier 0.080 ± 0.013). This consistency is reflected in Table 7, where DenseNet121 is ranked first and shows no major variance-driven weaknesses, indicating a predictable trade-off between missed metastasis and false alarms. In contrast, several models achieved excellent mean discrimination but displayed instability in clinically critical error dimensions. For example, DenseNet121-attention maintained top-tier discrimination (AUC-ROC 96.3 ± 1.1) yet exhibited higher variability in specificity (91.7 ± 9.2), implying that the false-alarm burden can fluctuate across folds, an issue explicitly highlighted in Table 7 as a risk for inconsistent follow-up recommendations. Similarly, DenseNet169 achieved high sensitivity (90.0 ± 3.8) but showed substantial variability in specificity (84.3 ± 11.7), suggesting that its clinical operating point can shift depending on the fold composition.

The most pronounced instability was observed in models with very large standard deviations (SDs), which raises concerns about reliability even when averages appear competitive. VGG19, despite very high sensitivity (92.7 ± 5.4), had extreme variability in specificity (69.3 ± 35.3), indicating highly erratic false-alarm behaviour across folds; accordingly, Table 7 characterises it as clinically risky due to unpredictable workload and decision consistency. The baseline CNN also showed marked instability, particularly in sensitivity (57.7 ± 29.5), implying unreliable detection of metastasis across folds. Variance was likewise substantial for the ResNet50 family, especially in sensitivity (ResNet50: 73.7 ± 21.5; ResNet50-attention: 62.0 ± 22.2), indicating inconsistent detection behaviour. Finally, ConvNeXt-Tiny exhibited catastrophic variability (sensitivity 46.7 ± 45.2; specificity 55.7 ± 46.1) alongside near-chance discrimination, confirming poor robustness and lack of clinical suitability in this setting. Finally, Table 4 and the stability-focused synthesis in Table 7 show that model selection should not be driven by peak AUC alone. Models such as DenseNet121 are preferable because they deliver high performance with low variance, whereas architectures with large fold-to-fold dispersion, especially in sensitivity or specificity, may yield clinically inconsistent missed metastasis or false-alarm behaviour across different patient subsets.

#### 4.2.5. Model Performance: Complexity (Efficiency and Deployability)

**Figure 8:**
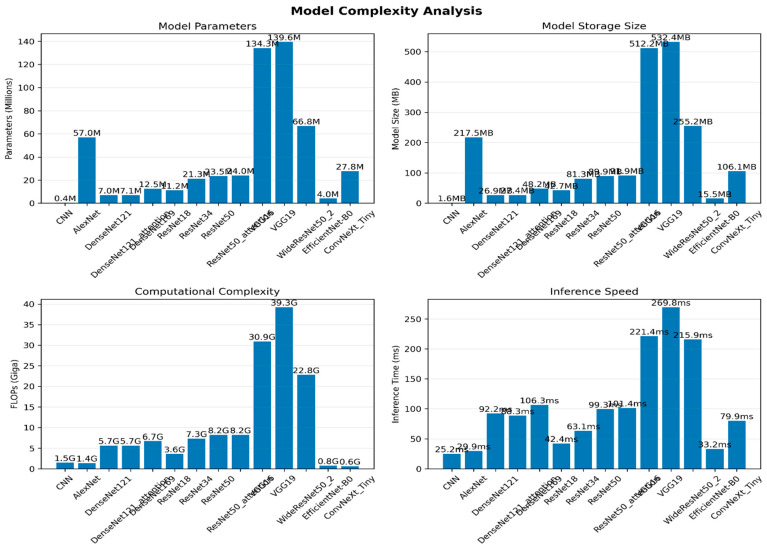
Model complexity.

In addition to diagnostic accuracy, clinical deployment requires models that are computationally feasible, especially in workflows where inference latency, memory footprint, and hardware availability are constrained. Therefore, Table 8 and Figure 8 compare each architecture in terms of parameter count, FLOPs, model size, and inference time, providing a practical view of efficiency–performance trade-offs. Overall, the results show that the highest-performing models are not always the most deployable, and conversely, the most efficient models may compromise detection reliability.

At the lightweight end, the baseline CNN was the most resource-efficient (0.4 M parameters, 1.5 G FLOPs, 1.6 MB, 25.2 ms), but its clinical utility was limited by weak and highly variable performance. EfficientNet-B0 provided a strong efficiency alternative, combining a small footprint (4.0 M parameters, 0.8 G FLOPs, 15.5 MB, 33.2 ms) with substantially better discrimination and specificity than the baseline, although its sensitivity remained lower than the top-performing DenseNet/VGG models. ResNet18 also offers a favourable speed profile (42.4 ms) with moderate model size (42.7 MB) and very high specificity, but at the cost of reduced sensitivity, which may be unacceptable in metastasis screening contexts.

The DenseNet models delivered the best accuracy–deployability compromise. DenseNet121 achieved top-tier predictive performance while remaining moderate in size (7.0 M parameters, 5.7 G FLOPs, 26.9 MB, 92.2 ms), and its attention variant was comparable (7.1 M, 5.7 G, 27.4 MB, 88.3 ms). Although slower than EfficientNet-B0 and ResNet18, these inference times remain within a feasible range for batch or workstation-based clinical reading. DenseNet169 increased complexity (12.5 M, 6.7 G, 48.2 MB, 106.3 ms) without a consistent improvement in performance, suggesting diminishing returns in exchange for higher computational cost.

In contrast, several architectures exhibited a heavy computational burden with limited incremental benefit. The VGG family was the least deployable, requiring very large parameter counts (134–140 M), extremely high FLOPs (30.9–39.3 G), massive storage footprints (512–532 MB), and the slowest inference times (221–270 ms), despite strong sensitivity. Likewise, WideResNet50_2 was computationally expensive (66.8 M, 22.8 G, 255.2 MB, 215.9 ms) relative to its performance tier. The ResNet50 variants were also considerably heavier than ResNet18/34 (~23–24 M parameters, 8.2 G FLOPs, ~90 MB, ~99–101 ms) yet did not surpass the DenseNet models. Finally, while ConvNeXt-Tiny appears relatively light in FLOPs (0.6 G), its model size (106.1 MB) and inference time (79.9 ms), combined with poor predictive performance, make it unsuitable for deployment under the current configuration.

Taken together, the complexity analysis indicates that DenseNet121 provides the most attractive overall deployment profile by pairing top-ranked performance with moderate computational requirements. EfficientNet-B0 and ResNet18 are viable when rapid inference and small footprints are prioritised, but their lower sensitivity may require threshold adjustment or calibration strategies to mitigate missed metastasis risk. Conversely, VGG16/VGG19 and WideResNet50_2 impose substantial computational cost that is difficult to justify given their limited gains relative to the best-performing DenseNet models.

#### 4.2.6. Summary of Findings

The comparative evaluation across 14 deep learning architectures indicates that DenseNet121 is the best overall model for detecting prostate cancer bone metastases from whole-body planar scintigraphy. It achieved the most balanced and consistently strong performance under stratified five-fold cross-validation, combining excellent discrimination (AUC-ROC 96.0 ± 1.2) with high accuracy (89.2 ± 2.2), strong F1-score (88.5 ± 2.5), and agreement (κ = 0.783 ± 0.045). Clinically, DenseNet121 offers a favourable reliability profile by maintaining high specificity (94.7 ± 2.2, low false-alarm burden) while sustaining robust sensitivity (83.7 ± 3.4, limited missed metastasis compared with conservative models). Importantly, it also produced the best-calibrated probabilities among all candidates (Brier score 0.080 ± 0.013), supporting trustworthy risk estimates for threshold-based decision support. Its superiority was reinforced by the statistical ranking analysis (best average rank under the significant Friedman test), and it showed minimal fold-to-fold variability relative to models with unstable sensitivity/specificity. From a deployment standpoint, DenseNet121 further provides a practical efficiency–performance trade-off (7.0 M parameters, 26.9 MB), making it feasible for routine clinical use without the heavy computational burden of VGG-style networks. Overall, DenseNet121 emerges as the most reliable and clinically suitable backbone for prostate cancer metastasis detection in bone scintigraphy, delivering the best combination of accuracy, robustness, calibration, and deployability.

## 5. Conclusions and Future Work

This study presented a comprehensive comparison of 14 deep learning architectures for automated detection of prostate cancer bone metastases from whole-body planar scintigraphy. Using a unified stratified five-fold cross-validation protocol, we evaluated discrimination, classification performance, clinical reliability (missed metastasis versus false alarms), calibration quality, stability across folds, and computational deployability. The results demonstrate clear architectural differences, supported by statistical testing, and highlight that model selection should consider not only peak accuracy but also robustness and clinical error trade-offs. Across all perspectives, DenseNet121 emerged as the most clinically suitable model. It achieved the best overall balance of performance and reliability, combining excellent discrimination (AUC-ROC 96.0 ± 1.2) with strong classification outcomes (accuracy 89.2 ± 2.2, F1-score 88.5 ± 2.5, κ = 0.783 ± 0.045) and the most favourable probability calibration (Brier score 0.080 ± 0.013). Clinically, DenseNet121 maintained high specificity (94.7 ± 2.2) while preserving robust sensitivity (83.7 ± 3.4), yielding a practical trade-off between minimising false alarms and limiting missed metastasis. Moreover, it exhibited low fold-to-fold variability and moderate computational cost, making it a strong candidate for deployment in real-world decision-support workflows. In contrast, high-sensitivity models (e.g., VGG variants) reduced missed metastasis but generated substantially higher and more variable false-alarm rates, while conservative models (e.g., ResNet18) minimised false alarms at the expense of increased missed metastasis risk.

Future work will focus on improving generalizability and clinical readiness in several directions. First, we will validate the proposed approach on multi-centre external datasets and across different scanners and acquisition protocols to quantify robustness under domain shift, and where feasible, we will also replicate the benchmark on publicly available bone scintigraphy datasets with compatible label definitions to provide additional external evidence prior to clinical deployment. Second, we will investigate probability calibration refinement (e.g., temperature scaling or isotonic regression) and threshold optimisation tailored to clinical objectives, enabling configurable operating points for screening-oriented versus workload-constrained settings. Third, we will extend the framework toward lesion-aware and region-guided learning, integrating anatomical priors or attention localisation to improve interpretability and to support clinician trust through visual explanations by integrating XAI evaluation. Fourth, we will explore semi-supervised and self-supervised pretraining to reduce reliance on labelled data and improve feature transferability in limited-data settings. Finally, we will optimise deployment through model compression and acceleration (quantisation, pruning, and knowledge distillation) and conduct prospective studies to evaluate workflow integration, runtime performance, and real-world clinical impact. Overall, the findings indicate that DenseNet-based representations, particularly DenseNet121, provide an effective and deployable foundation for prostate cancer bone metastasis detection from planar scintigraphy, and the proposed future directions will further strengthen robustness, interpretability, and clinical translation. The main limitations of this study are: (1) single-centre retrospective design, which may limit generalizability across institutions and equipment manufacturers; (2) absence of external multi-institutional validation; (3) binary classification without metastatic burden stratification (e.g., Soloway classification) or osteoblastic/osteolytic subtyping; (4) reference standard based on composite clinical follow-up rather than uniform histopathological confirmation or PSMA PET/CT; (5) lack of lesion-level annotations precluding localization performance assessment; and (6) data sharing restricted by institutional governance, preventing independent replication.

## Figures and Tables

**Table 1:** Summary of related studies.

Study ID	Modality/Task	DL Architecture	Dataset (Approx.)	Validation Evaluation	Reported Performance	Limitations
[31]	Prostate MRI; cancer vs. non-cancer classification	Transfer-learning GoogleNet CNN vs. classical ML	Public Harvard MRI dataset (prostate + brachytherapy cases)	Train/test split with comparison to SVM, DT, NB, etc.	CNN markedly outperformed handcrafted-feature ML; reported 100% sensitivity, specificity, and AUC on their test set	Single backbone and single dataset; small sample size; no cross-validated or multi-architecture benchmark
[32]	Prostate MRI; PCa detection	ResNet-101 (transfer learning) and LSTM vs. several classical classifiers	Public MRI dataset; few hundred cases	10-fold CV with multiple metrics (ACC, SEN, SPE, MCC, AUC)	ResNet-101 achieved near-perfect accuracy and AUC (~1.0), outperforming optimised GLCM + classical ML	Only one deep residual model; MRI-only; high results on a limited dataset may not generalise
[33]	Bi-parametric MRI; clinically significant PCa detection and localisation	Cascaded 3D U-Net/detection U-Net with zonal priors	2734 biopsy-naïve patients (multi-institutional)	Internal CV + external test set; lesion- and patient-level metrics	External-test AUC 0.85; lesion sensitivity 87% at 1 FP/patient	Shows the benefit of large data and anatomical priors; still a single architecture family and MRI-only
[34]	Planar whole-body bone scintigraphy; bone metastasis vs. non-metastasis	Lightweight 2D CNNs: WB model and GLUE (global–local)	9133 scans from 5342 PCa patients	Cross-validation under abundant vs. limited data	AUC 0.933–0.957 (WB) and 0.936–0.955 (GLUE) with abundant data; GLUE superior with limited data	Focused on custom lightweight CNNs; no systematic comparison of standard backbones (ResNet, VGG, DenseNet, etc.)
[35]	Bi-parametric MRI; lesion detection (ISUP ≥ 1)	3D U-Net vs. AH-Net	525 patients from two institutions	Internal + external cohorts; lesion and patient level	Patient-level sensitivity > 90%; AH-Net lesion sensitivity 74.4% with fewer FPs	Strong multi-centre design, but limited to two 3D architectures; computational aspects not analysed
[36]	Bi-parametric MRI; csPCa detection	Report-Guided SSL (RG-SSL) using CNN backbone	7756 exams from 6380 patients + external 300-patient set	Semi-supervised training; comparison with fully supervised and other SSL methods	AUC 0.86 ± 0.01 using only 100 labelled exams; performance comparable to models trained on >3000 labelled cases	Demonstrates the power of weak supervision; depends on structured reports; MRI-only, with a single backbone class
[37]	Prostate MRI; lesion detection and localization	Modified ResNet50 + Faster R-CNN/Mask R-CNN (PCDM)	~11,000 MRI images	80/20 train–test split; ablation studies	Accuracy 95.24%, sensitivity 97.40%, specificity 97.09%, precision 97.56%	Custom hybrid architecture; single modality; no comparison across multiple standard CNN families under identical settings
[38]	[^18^F]-PSMA-1007 PET/CT; local recurrence detection	DenseNet121-based CNN variants (whole-body/cropped/metadata-enhanced)	1404 PET/CT exams from 1145 patients	Internal train/val/test split with external testing	Best model: validation accuracy 77.1%, balanced accuracy 73.9%, AUC 0.75; sensitivity dropped on external test	Provides important negative evidence; limited performance and signs of overfitting; the dataset is still relatively modest for a complex PET/CT task

**Table 2:** Training configuration hyperparameter.

Category	Hyperparameter	Value (as Used in Code)
Input & Preprocessing	Input image size	224 × 224
	Colour mode	RGB
	Normalisation means	[0.485, 0.456, 0.406]
	Normalisation std	[0.229, 0.224, 0.225]
Data Augmentation (Train Only)	Random horizontal flip	*p* = 0.5
	Random rotation	±10°
	Colour jitter	Brightness = 0.2, Contrast = 0.2
Cross-Validation	Outer CV strategy	Stratified K-Fold
	Number of folds	5
	Train/Val split (within each fold)	80%/20% (stratified)
Optimisation	Loss function	CrossEntropyLoss
	Optimiser	AdamW
	Learning rate	1 × 10^−4^
	Weight decay	1 × 10^−4^
Training	Batch size	16
	Max epochs	50
	Early stopping monitor	Validation ROC–AUC
	Early stopping patience	10 epochs
Reproducibility	Random seed	42
Output Layer	Number of output logits	2 (Normal vs. Abnormal)
Uncertainty (Reported)	Confidence level	95%
	Bootstrap samples (per metric)	1000
Number of Runs	Run repeated	10

**Table 3:** CNN model configuration hyperparameter.

Model Name	Architectural Family	Backbone Design	Key Connectivity	Attention Module	Binary Classification Head Adaptation
CNN	Custom/Classical	4-block CNN (Conv → BN → ReLU → MaxPool) with channels 32 → 64 → 128 → 256	Feed-forward	None	AdaptiveAvgPool → MLP: 256 → 128 → 2 with Dropout (0.5, 0.3)
AlexNet	Classical	AlexNet-style conv stack + pooling	Feed-forward	None	AdaptiveAvgPool (6 × 6) → (256 × 6 × 6) → 4096 → 4096 → 2 with Dropout (0.5)
VGG16	VGG Family	Deep sequential 3 × 3 conv blocks	Feed-forward	None	Replace final FC: 4096 → 2
VGG19	VGG Family	Deeper VGG (more conv layers)	Feed-forward	None	Replace final FC: 4096 → 2
ResNet18	Residual Network	Basic residual blocks	Residual skip connections	None	GlobalAvgPool → 512 → 2
ResNet34	Residual Network	Deeper basic residual blocks	Residual skip connections	None	GlobalAvgPool → 512 → 2
ResNet50	Residual Network	Bottleneck residual blocks	Residual skip connections	None	GlobalAvgPool → 2048 → 2
ResNet50_attention	Residual Network	Bottleneck residual blocks	Residual skip connections	CBAM (2048) before avgpool	GlobalAvgPool → 2048 → 2
DenseNet121	Dense Network	Dense blocks + transitions (121 layers)	Dense connectivity (feature concatenation)	None	GlobalAvgPool → 1024 → 2
DenseNet121_attention	Dense Network	Dense blocks + transitions (121 layers)	Dense connectivity (feature concatenation)	CBAM (1024) before pooling	GlobalAvgPool → 1024 → 2
DenseNet169	Dense Network	Deeper dense configuration (169 layers)	Dense connectivity (feature concatenation)	None	GlobalAvgPool → 1664 → 2
WideResNet50_2	Residual Network	Wide bottleneck blocks	Residual skip connections (wider channels)	None	GlobalAvgPool → 2048 → 2
EfficientNet-B0	EfficientNet	MBConv blocks	Compound scaling baseline	Built-in SE (within MBConv)	Replace classifier: 1280 → 2
ConvNeXt-Tiny	Modern CNN	ConvNeXt blocks (modernised conv design)	Stage-wise hierarchical conv	None	Replace classifier: 768 → 2

**Table 4:** Obtained quantitative performance results.

Model	AUC-ROC	Sensitivity	Specificity	Accuracy	Precision	F1-Score	Kappa	Brier Score
CNN	89.0 ± 2.3	57.7 ± 29.5	88.7 ± 16.2	73.2 ± 11.5	89.9 ± 11.8	62.6 ± 28.4	0.463 ± 0.230	0.172 ± 0.048
AlexNet	94.8 ± 0.4	82.7 ± 9.8	90.3 ± 2.9	86.5 ± 3.7	89.8 ± 2.0	85.6 ± 5.0	0.730 ± 0.073	0.100 ± 0.018
DenseNet121	96.0 ± 1.2	83.7 ± 3.4	94.7 ± 2.2	89.2 ± 2.2	94.0 ± 2.5	88.5 ± 2.5	0.783 ± 0.045	0.080 ± 0.013
DenseNet121_attention	96.3 ± 1.1	85.3 ± 3.6	91.7 ± 9.2	88.5 ± 3.0	92.1 ± 7.4	88.3 ± 2.2	0.770 ± 0.061	0.085 ± 0.017
DenseNet169	96.0 ± 1.1	90.0 ± 3.8	84.3 ± 11.7	87.2 ± 4.2	86.4 ± 8.3	87.7 ± 3.0	0.743 ± 0.083	0.093 ± 0.033
ResNet18	93.9 ± 1.3	63.7 ± 12.9	97.3 ± 2.3	80.5 ± 5.9	96.2 ± 2.7	75.7 ± 10.1	0.610 ± 0.119	0.144 ± 0.035
ResNet34	94.7 ± 1.3	85.0 ± 3.2	89.0 ± 7.0	87.0 ± 4.2	88.9 ± 6.2	86.8 ± 3.9	0.740 ± 0.084	0.092 ± 0.014
ResNet50	90.8 ± 6.3	73.7 ± 21.5	82.7 ± 15.6	78.2 ± 7.3	83.7 ± 9.0	75.5 ± 12.9	0.563 ± 0.145	0.154 ± 0.049
ResNet50_attention	89.3 ± 5.7	62.0 ± 22.2	93.3 ± 5.6	77.7 ± 9.1	92.3 ± 5.8	70.6 ± 18.9	0.553 ± 0.182	0.144 ± 0.053
VGG16	94.1 ± 3.5	91.3 ± 1.9	78.0 ± 9.7	84.7 ± 5.3	81.1 ± 7.0	85.8 ± 4.3	0.693 ± 0.106	0.107 ± 0.034
VGG19	93.6 ± 3.5	92.7 ± 5.4	69.3 ± 35.3	81.0 ± 15.8	80.2 ± 16.1	84.6 ± 9.3	0.620 ± 0.315	0.122 ± 0.067
WideResNet50_2	94.6 ± 1.2	76.7 ± 10.7	95.7 ± 4.0	86.2 ± 3.9	95.3 ± 3.9	84.3 ± 5.8	0.723 ± 0.078	0.105 ± 0.033
EfficientNet-B0	89.2 ± 4.5	68.3 ± 13.0	92.3 ± 5.3	80.3 ± 5.3	90.6 ± 5.0	76.9 ± 9.0	0.607 ± 0.106	0.139 ± 0.031
ConvNeXt_Tiny	52.0 ± 9.3	46.7 ± 45.2	55.7 ± 46.1	51.2 ± 2.3	32.1 ± 26.5	35.3 ± 30.1	0.023 ± 0.047	0.250 ± 0.002

**Table 5:** Clinical reliability.

Model	Sensitivity (%)	FNR (%)	Specificity (%)	FPR (%)	Clinical Risk Profile
VGG19	92.7 ± 5.4	7.3 ± 5.4	69.3 ± 35.3	30.7 ± 35.3	Lowest missed metastasis; highest false-alarm rate; suitable for high-stakes screening
VGG16	91.3 ± 1.9	8.7 ± 1.9	78.0 ± 9.7	22.0 ± 9.7	Very high sensitivity; moderate false alarms; reliable for metastasis detection
DenseNet169	90.0 ± 3.8	10.0 ± 3.8	84.3 ± 11.7	15.7 ± 11.7	Excellent sensitivity; balanced specificity; strong clinical performer
ResNet34	85.0 ± 3.2	15.0 ± 3.2	89.0 ± 7.0	11.0 ± 7.0	Good balance; moderate missed cases; acceptable false-alarm rate
DenseNet121-attention	85.3 ± 3.6	14.7 ± 3.6	91.7 ± 9.2	8.3 ± 9.2	Enhanced sensitivity with attention; variable specificity across folds
AlexNet	82.7 ± 9.8	17.3 ± 9.8	90.3 ± 2.9	9.7 ± 2.9	Surprisingly robust; good specificity; moderate sensitivity
DenseNet121	83.7 ± 3.4	16.3 ± 3.4	94.7 ± 2.2	5.3 ± 2.2	Optimal clinical balance; low false alarms with strong detection
WideResNet50_2	76.7 ± 10.7	23.3 ± 10.7	95.7 ± 4.0	4.3 ± 4.0	Excellent specificity; moderate sensitivity; low false-alarm burden
ResNet50	73.7 ± 21.5	26.3 ± 21.5	82.7 ± 15.6	17.3 ± 15.6	Variable performance; high uncertainty in both metrics
CNN (Baseline)	57.7 ± 29.5	42.3 ± 29.5	88.7 ± 16.2	11.3 ± 16.2	Highly variable; poor sensitivity; moderate specificity
EfficientNet-B0	68.3 ± 13.0	31.7 ± 13.0	92.3 ± 5.3	7.7 ± 5.3	Good specificity; suboptimal sensitivity; efficient but limited detection
ResNet50-attention	62.0 ± 22.2	38.0 ± 22.2	93.3 ± 5.6	6.7 ± 5.6	High specificity; poor sensitivity; attention reduces false alarms
ResNet18	63.7 ± 12.9	36.3 ± 12.9	97.3 ± 2.3	2.7 ± 2.3	Lowest false alarms; high missed metastasis; conservative classifier
ConvNeXt_Tiny	46.7 ± 45.2	53.3 ± 45.2	55.7 ± 46.1	44.3 ± 46.1	Poor performance; near random

**Table 6:** Discrimination vs. calibration performance.

Model	AUC-ROC	Discrimination Category	Brier Score	Calibration Category	Combined Performance
DenseNet121-attention	0.963 ± 0.011	Excellent	0.085 ± 0.017	Excellent	Best overall
DenseNet121	0.960 ± 0.012	Excellent	0.080 ± 0.013	Optimal	Optimal balance
DenseNet169	0.960 ± 0.011	Excellent	0.093 ± 0.033	Good	Excellent
AlexNet	0.948 ± 0.004	Excellent	0.100 ± 0.018	Good	Strong
ResNet34	0.947 ± 0.013	Excellent	0.092 ± 0.014	Good	Strong
WideResNet50_2	0.946 ± 0.012	Excellent	0.105 ± 0.033	Moderate	Good
VGG16	0.941 ± 0.035	Good	0.107 ± 0.034	Moderate	Good
ResNet18	0.939 ± 0.013	Good	0.144 ± 0.035	Poor	Discordant
VGG19	0.936 ± 0.035	Good	0.122 ± 0.067	Moderate	Moderate
ResNet50	0.908 ± 0.063	Moderate	0.154 ± 0.049	Poor	Weak
EfficientNet-B0	0.892 ± 0.045	Moderate	0.139 ± 0.031	Poor	Weak
ResNet50-attention	0.893 ± 0.057	Moderate	0.144 ± 0.053	Poor	Weak
CNN (Baseline)	0.890 ± 0.023	Moderate	0.172 ± 0.048	Very poor	Poor
ConvNeXt-Tiny	0.520 ± 0.093	Chance-level	0.250 ± 0.002	Very poor	Clinically unacceptable

Category thresholds: Discrimination (AUC): Excellent ≥ 0.95, good 0.90–0.95, moderate 0.85–0.90, chance level ≈ 0.50. Calibration (Brier): Excellent/optimal ≤ 0.10, good 0.10–0.15, moderate 0.15–0.20, very poor > 0.20.

**Table 7:** Performance stability analysis (5-fold CV).

Rank	Model	Avg Rank	Key Strength (Mean ± SD)	Critical Weakness (Variance)	Clinical Risk
1	DenseNet121	3.0	Best overall stability among top performers (AUC-ROC 96.0 ± 1.2, Sens 83.7 ± 3.4, Spec 94.7 ± 2.2)	Nonsignificant (consistently low SD across metrics)	Lowest risk; predictable, high-performance behaviour
2	DenseNet121-attention	3.6	Highest discrimination (AUC-ROC 96.3 ± 1.1) with strong sensitivity (85.3 ± 3.6)	High variance in specificity (91.7 ± 9.2)	Unstable false-alarm rate; inconsistent false positives across folds
3	DenseNet169	3.8	High sensitivity (90.0 ± 3.8) with excellent AUC (96.0 ± 1.1)	Very high variance in specificity (84.3 ± 11.7)	Unreliable trade-off; false-alarm burden shift across folds
4	VGG16	5.4	Very high sensitivity (91.3 ± 1.9) and strong F1 (85.8 ± 4.3)	Notable variance in specificity (78.0 ± 9.7)	Higher false-alarm burden; more follow-ups expected
5	ResNet34	5.8	Balanced profile (Sens 85.0 ± 3.2, Spec 89.0 ± 7.0) with strong AUC (94.7 ± 1.3)	Moderate variability in specificity (±7.0)	Moderate risk; trade-off shifts moderately across folds
6	WideResNet50_2	6.0	Excellent specificity (95.7 ± 4.0) and precision (95.3 ± 3.9)	Higher variance in sensitivity (76.7 ± 10.7)	Missed-metastasis risk can vary; detection is less consistent
7	AlexNet	6.6	Extremely stable AUC (94.8 ± 0.4) and stable specificity (90.3 ± 2.9)	Higher variance in sensitivity (82.7 ± 9.8)	Inconsistent detection rate; misses can fluctuate
8	VGG19	7.0	Very high sensitivity (92.7 ± 5.4)	Extreme variance in specificity (69.3 ± 35.3)	Highly erratic false-alarm behaviour; unreliable workload impact
9	ResNet18	8.4	Highest specificity tier (97.3 ± 2.3) → very low false alarms	Lower/variable sensitivity (63.7 ± 12.9)	Conservative classifier; increased missed-metastasis risk
10	ResNet50	8.8	No dominant stable advantage; moderate overall	Very high variance in sensitivity (73.7 ± 21.5) and specificity (82.7 ± 15.6)	Clinically unstable; both misses and alarms fluctuate
11	EfficientNet-B0	10.4	Efficient model with good specificity (92.3 ± 5.3)	Higher variance in sensitivity (68.3 ± 13.0)	Moderate missed-metastasis risk; detection stability limited
12	ResNet50-attention	11.0	High specificity (93.3 ± 5.6) → fewer false alarms on average	Extreme variance in sensitivity (62.0 ± 22.2)	High + unstable missed-metastasis risk; unreliable detection
13	CNN (Baseline)	11.2	Lightweight; moderate average specificity (88.7 ± 16.2)	Extreme variance in sensitivity (57.7 ± 29.5)	Highly erratic; fails to detect metastasis consistently
14	ConvNeXt-Tiny	14.0	—	Catastrophic variance (Sens 46.7 ± 45.2, Spec 55.7 ± 46.1) + near-chance AUC (52.0 ± 9.3)	Clinically unacceptable; unstable and near-random

**Table 8:** Complexity (efficiency and deployability).

Model	Params (M)	FLOPs (G)	Model Size (MB)	Inference (ms)
CNN	0.4	1.5	1.6	25.2
AlexNet	57.0	1.4	217.5	29.9
DenseNet121	7.0	5.7	26.9	92.2
DenseNet121_attention	7.1	5.7	27.4	88.3
DenseNet169	12.5	6.7	48.2	106.3
ResNet18	11.2	3.6	42.7	42.4
ResNet34	21.3	7.3	81.3	63.1
ResNet50	23.5	8.2	89.9	99.3
ResNet50_attention	24.0	8.2	91.9	101.4
VGG16	134.3	30.9	512.2	221.4
VGG19	139.6	39.3	532.4	269.8
WideResNet50_2	66.8	22.8	255.2	215.9
EfficientNet-B0	4.0	0.8	15.5	33.2
ConvNeXt_Tiny	27.8	0.6	106.1	79.9

## Data Availability

The data supporting the findings of this study are available from the corresponding author upon reasonable request. The data are not publicly available due to privacy and ethical restrictions.
